# Detection of MLV-like gag sequences in blood samples from a New York state CFS cohort

**DOI:** 10.1186/1742-4690-8-S1-A234

**Published:** 2011-06-06

**Authors:** Maureen R Hanson, Li L Lee, Lin Lin, David E Bell, David Ruppert, David S Bell

**Affiliations:** 1 Dept. of Molecular Biology and Genetics, Cornell University, Ithaca, NY, 14853, USA; 2 Dept. of Medical Anthropology, State University of New York, Buffalo, NY, 14214, USA; 3 School of Operations Research and Information Engineering, Cornell University, Ithaca, NY, 14853, USA; 4 Dept. of Pediatrics, State University of New York, Buffalo, NY, 14214, USA

## 

A blinded study was undertaken to determine whether XMRV or MLV-like virus could be detected in peripheral blood from 40 adult subjects divided into three groups: severely ill with CFS, recovered from CFS, and a control group lacking a CFS diagnosis at any time. All patients in the“severe CFS” group currently meet Fukuda criteria. “Recovered CFS” subjects had scores on the SF-36 survey instrument that were significantly lower than the healthy control group, according to Hotelling’s T2 test. Blood was collected in EDTA tubes and cDNA and DNA made from PBMCs. Plasma was incubated with LNCaP cells that were subsequently passaged. Nested PCR with USB Hot-Start IT FideliTaq was performed with gag primers. Any PCR products of expected sizes were sequenced. Samples were tested for mouse contamination with primers to IAP and/or mouse mitochondrial DNA. gag sequences were detected in both severe and recovered CFS subjects’ blood as well as in some healthy controls. gag sequences could be amplified from genomic DNA from LNCaP cells of some subjects after 4 or 6 subcultures following incubation with certain subjects’ plasma, indicating the presence of infectious virus in blood. All gag sequences detected in this cohort were more similar to the MLV-like sequences reported by Lo et al. (2010) than to the XMRV sequences reported by Lombardi et al. (2009). Detection of gag sequences in whole blood genomic DNAs that were negative for mouse IAP and mitochondrial DNA provides strong evidence for infection of humans with MLV-like viruses.

